# Nephrologists Hate the Dialysis Catheters: A Systemic Review of Dialysis Catheter Associated Infective Endocarditis

**DOI:** 10.1155/2017/9460671

**Published:** 2017-03-20

**Authors:** Kalyana C. Janga, Ankur Sinha, Sheldon Greenberg, Kavita Sharma

**Affiliations:** ^1^Department of Nephrology, Maimonides Medical Center, Brooklyn, NY, USA; ^2^Department of Internal Medicine, Maimonides Medical Center, Brooklyn, NY, USA; ^3^Department of Infectious Diseases, Maimonides Medical Center, Brooklyn, NY, USA

## Abstract

A 53-year-old Egyptian female with end stage renal disease, one month after start of hemodialysis via an internal jugular catheter, presented with fever and shortness of breath. She developed desquamating vesiculobullous lesions, widespread on her body. She was in profound septic shock and broad spectrum antibiotics were started with appropriate fluid replenishment. An echocardiogram revealed bulky leaflets of the mitral valve with a highly mobile vegetation about 2.3 cm long attached to the anterior leaflet. CT scan of the chest, abdomen, and pelvis showed bilateral pleural effusions in the chest, with triangular opacities in the lungs suggestive of infarcts. There was splenomegaly with triangular hypodensities consistent with splenic infarcts. Blood cultures repeatedly grew* Candida albicans*. Despite parenteral antifungal therapy, the patient deteriorated over the course of 5 days. She died due to a subsequent cardiac arrest. Systemic review of literature revealed that the rate of infection varies amongst the various types of accesses, and it is well documented that AV fistulas have a much less rate of infection in comparison to temporary catheters. All dialysis units should strive to make a multidisciplinary effort to have a referral process early on, for access creation, and to avoid catheters associated morbidity.

## 1. Introduction

End stage renal disease (ESRD) is rampant in the population today. As per the data reported in the US renal data reporting system by the national institutes of health (NIH), the number of patients being treated by hemodialysis is at a record high [1]. They reported 678,383 prevalent cases of ESRD, and the number continues to rise by about 21000 cases per year. The onus falls on the nephrologists to provide safe and good quality dialysis, while the patients wait for their transplant. This includes getting appropriate vascular access for performing the procedure.

Infective endocarditis is an infection of the endocardial layer of the heart. Patients receiving chronic hemodialysis are at an increased risk of infective endocarditis [2] and the disease course is protracted with significant mortality and morbidity [3]. It is a well known fact that temporary vascular access in the form of central lines and dialysis catheters further increase the risk of life threatening bacteremia and in turn infective endocarditis.

We report a case of severe endocarditis with marked complications associated with a temporary vascular access in a patient recently commenced on dialysis. We aim to shed light on the pitfalls of continuing dialysis on such forms of vascular accesses and to review the diagnosis and management of infective endocarditis in similar cases.

## 2. Case Presentation

A 53-year-old Egyptian female with past medical history of hypertension with nephropathy leading to end stage renal disease, one month after the start of hemodialysis, presented with fever and shortness of breath at an Egyptian hospital. Blood cultures grew* Staphylococcus aureus* and the sepsis was treated aggressively with piperacillin with tazobactam and Imipenem. She was transported from Egypt for further treatment in the United States.

On presentation at the referral center the patient was found to be in septic shock.

**Figure 1:**
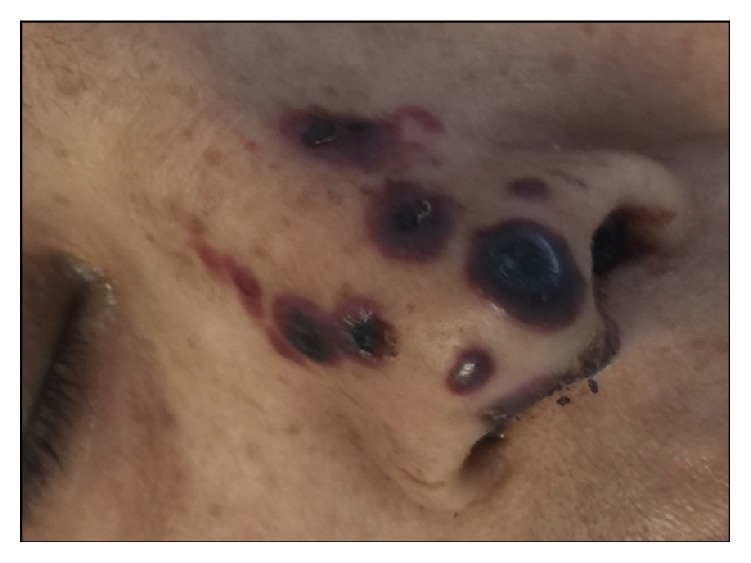
Image depicting severe end arteriolar embolic phenomenon to the nose.

**Figure 2:**
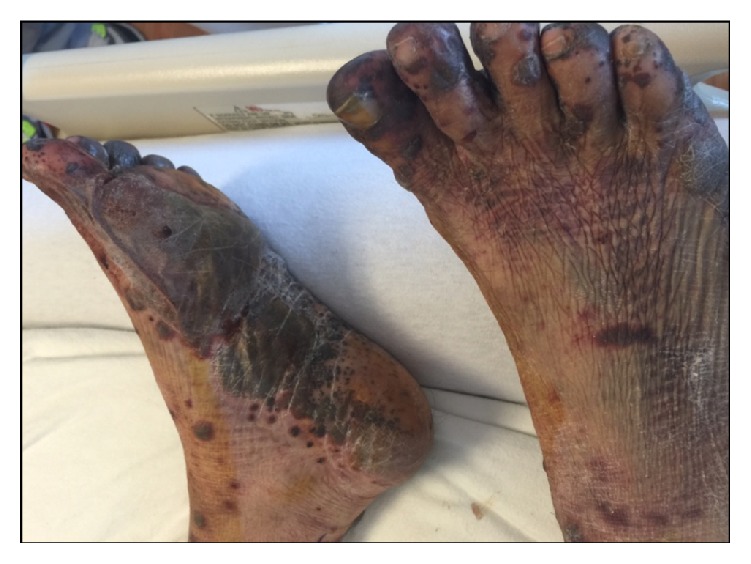
Image depicting desquamating vesiculobullous lesions of the feet.

Her blood pressure on admission was 73/41 mm of mercury, with a heart rate of 120 beats per minute and a respiratory rate of 22 breaths per minute. Her white cell count on admission was 9,400 cells/*μ*L; the differential count included 97.9% neutrophils with 2% lymphocytes. C-reactive protein on admission was 4.7 mg/dL. She was hypotensive and tachycardic. She had vesiculobullous lesions on the nose (Figure 1), forearms, and the feet (Figure 2). Some of the lesions desquamated to leave ulcers. She had a previously placed left sided internal jugular central line for hemodialysis. This central line was immediately removed on presentation and a fresh dialysis catheter was placed. Blood cultures grew* Candida albicans* on day 1 and continued to grow in all blood culture bottles consistently, during her stay. Appropriate investigations for infective endocarditis were performed.

**Figure 3:**
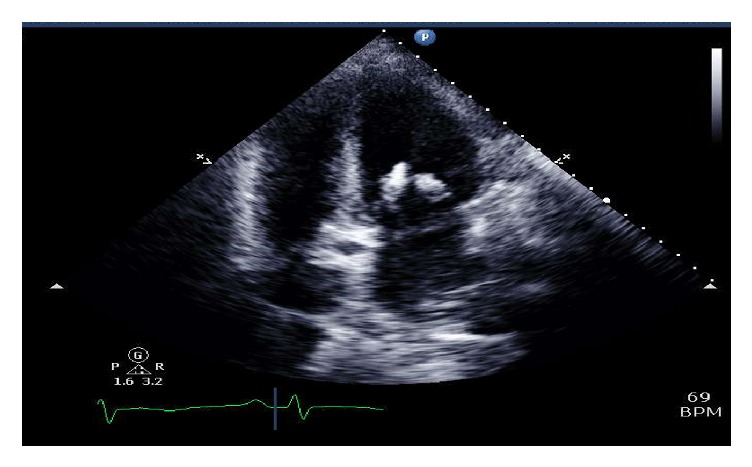
Image depicting a transthoracic echo cardiogram, depicting vegetation and severe mitral regurgitation.

**Figure 4:**
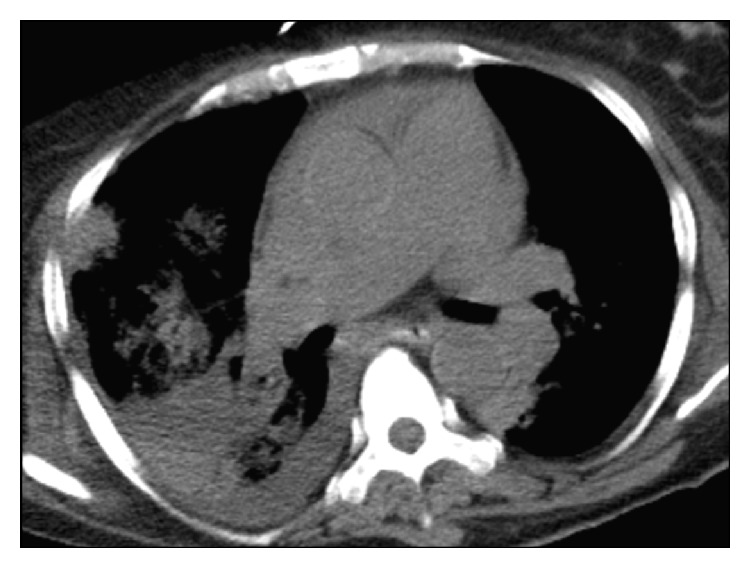
CT scan of the chest, depicting wedge shaped large pulmonary infarct.

**Figure 5:**
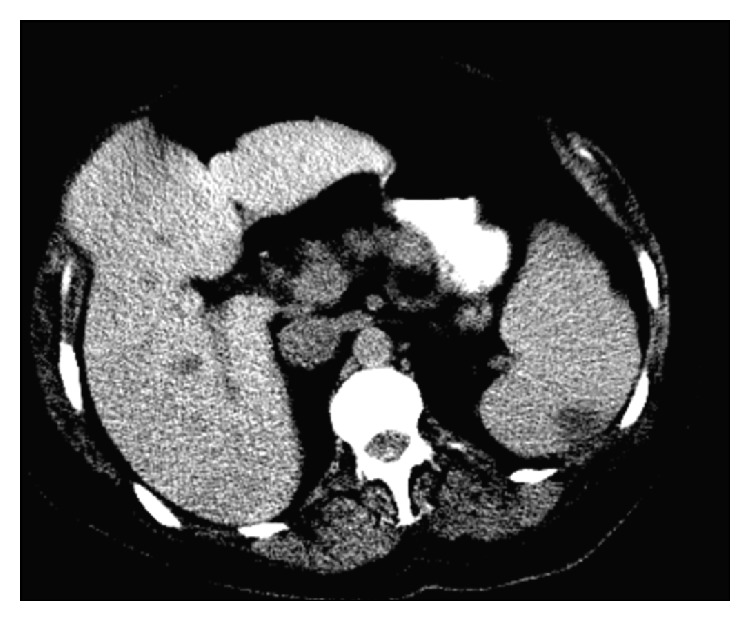
CT scan of the abdomen, depicting splenic infarct.

A transthoracic echocardiogram revealed bulky leaflets of the mitral valve with a highly mobile vegetation about 2.3 cm long attached to the anterior leaflet (Figure 3). This vegetation was prolapsing into the left atrium and was causing moderate mitral regurgitation. Computed tomogram (CT) scan of the chest, abdomen, and pelvis was also performed. It showed bilateral pleural effusions in the chest, with triangular opacities in the lungs suggestive of infarcts (Figure 4). There was mild splenomegaly with triangular hypodensities consistent with splenic infarcts (Figure 5). A CT scan of the abdomen and pelvis was found to appropriately visualize the renal system; there were atrophic kidneys bilaterally, with no evidence of stones. The bladder was collapsed on the scan.

Despite initiating parenteral antifungal therapy, the patient deteriorated over the course of 5 days. Her disease progressed to cause multiple organ failure and she was placed on palliative care due to grave prognosis and to honor the family's wishes. She died due to a cardiac arrest.

## 3. Discussion

### 3.1. Microbiology

The center for disease control and prevention (CDC) issued a dialysis surveillance report with data for participating centers the United States. This report utilized the CDC's national health safety network (NHSN) for reporting facts about patients receiving hemodialysis. This network involved reporting of adverse events associated with dialysis and analyzing the data. Out of the 599 bacterial isolates from the 532 positive blood cultures following an adverse event, 77% (461 isolates) were associated with central lines. Although common skin contaminants took a major chunk of these isolates (44.3%),* Staphylococcus aureus* also represented major causation (19.7%). It is also concerning to note that there is a stark difference in the rate of bacteremia in temporary lines in comparison to patients with a graft or arteriovenous fistula (138 isolates comprising of 17%) [4]. 42% of all reported isolates of Staphylococcus aureus were MRSA.

It is interesting to note that fungal infections leading to endocarditis, similar to our patient, comprised of a mere 1.7% in Central line associated infections and 2.9% in fistula or graft associated infections.

### 3.2. Predisposing Factors

**Figure 6:**
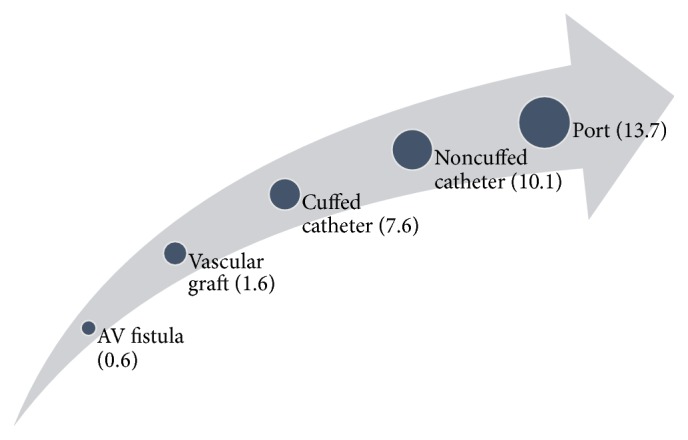
Depicting vascular access infection rate by type of vascular access.

Strom et al. reported a 16.9% relative risk of IE in hemodialysis patients in comparison to the general population [5]. One of the most important factors is the propensity of having bacteremia in patients needing HD. These frequent episodes of bacteremia can be attributed to repeated IV access through vascular catheters, grafts, and fistulas [6]. The rate of infection varies amongst the various types of access, and it is well documented that AV fistulas have a much lower rate of infection in comparison to temporary catheters.  Figure 6 depicts the rate of vascular access infection as per a report by the CDC [7]. This theory is confirmed by the fact that rate of endocarditis is less in patients getting peritoneal dialysis in comparison to general population [8]. While the patients with peritoneal dialysis have lower rates of infection than hemodialysis their rates of infective endocarditis are still higher than general population [3].

There is literature suggesting salvage of central lines if it is technically unfeasible to remove the lines. The suggested measures include antibiotic lock therapy with varying concentrations of solutions like antibiotics [9], ethanol [10], and nitroglycerin [10]. The consensus is to remove the infected line unless an extenuating circumstance prevents removing the line.

Some practices have been suggested to decrease the rate of infections associated with central lines; these include use of chlorhexidine impregnated dressings, catheter care with chlorhexidine solution, and dressing changes every 5–7 days. Catheter hubs and ports should be cleaned with either 70% alcohol or chlorhexidine [11].

Patients with ESRD have an increased incidence of heart valve disease. The valve disorders are secondary to calcification leading to regurgitation or stenosis [12]. The onset of valve disease in HD patients is earlier by 10–20 years in comparison to general population. This has been attributed to abnormal calcium and phosphorus metabolism, secondary to hyperparathyroidism [13].

**Figure 7:**
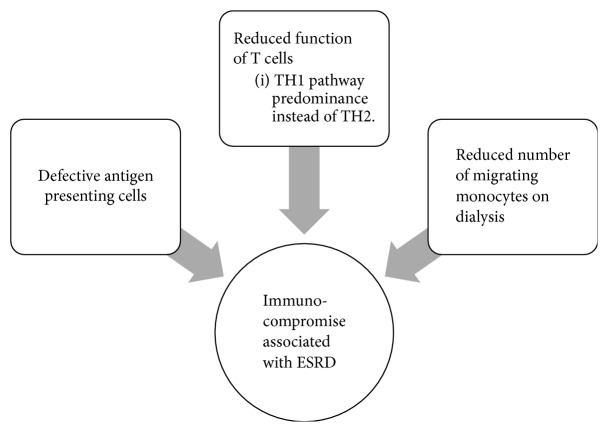
Pathophysiology of impeded immune function in renal failure.

ESRD results in an impaired immune status. This is multifactorial and includes a defect in antigen presenting cells. This leads to reduced stimulation of T Lymphocytes. This has been well documented and was originally observed due to decreased response to hepatitis vaccine in patients with ESRD [14]. T cell activation is hampered in patient with ESRD, and there is a predisposition of T Helper Cell 1 (TH1) pattern instead of T Helper 2 (TH2) pattern. This leads to a decrease in amount of antibody production [15]. There is a decrease in the number of circulating monocytes on starting HD. This is secondary to their activation on coming in contact with the dialyzing membrane and a state of chronic inflammation [16], although it is yet to be proven if this has any effect on the decreased immune response.  Figure 7 depicts the pathophysiology of impaired immune function in renal failure.

### 3.3. Diagnosis

The diagnosis of infective endocarditis is specifically challenging in patients on HD. Duke's criterion is most trusted for predicting and diagnosing infective endocarditis, but applying the criterion to patient with ESRD on HD is tricky. Duke's criteria have major and minor criteria; one of the key major criteria is two positive blood cultures with an organism consistent with infective endocarditis, in the absence of a focus of infection. The frequent presence of a plausible source of infection, in the form of central lines, or ports can often make it difficult to differentiate between endocarditis and an uncomplicated line infection [17]. Similarly, fever is a minor criterion in Duke's Criteria. Patients with ESRD have a flattened immune response, and they do not mount fevers as effectively as the general population [2].

Thus echocardiography in the presence of a high suspicion of endocarditis in a patient with HD can be life-saving. Like any other patient with suspected infective endocarditis the initial imaging modality is a transthoracic echocardiogram, followed by a transesophageal echocardiogram if the image quality is questionable. Another indication of a transesophageal echocardiogram would be high suspicion even after a transthoracic study negates the diagnosis. Gaetano et al. published some high suspicion features mandating transesophageal study; these features are depicted in figure. They recommend a mandatory TEE with a TTE if any of these features are present (Table 1).

### 3.4. Treatment

The treatment of IE involves prompt diagnosis with the start of empiric therapy. After blood cultures have isolated an organism, a more directed approach can be followed.  Table 2 depicts the appropriate therapy for empiric as well as organism specific therapy as per published guidelines for American family physicians.

Special consideration should be made in patients with ESRD and vancomycin should be avoided for methicillin susceptible* S. aureus* as it has lower bactericidal action in comparison to oxacillin or cefazolin. It also contributes to selection of S aureus strains with reduced sensitivity to glycopeptides and vancomycin resistant enterococci [18]. Vancomycin remains the first choice for MRSA infections. Due to the rising rate of* S. aureus* strains with increased minimal inhibitory concentration of vancomycin, drugs like daptomycin and linezolid should be considered [19].

Candida endocarditis is a rare entity in native valves, and the risk of having a fungal infection causing endocarditis rises in the immunocompromised, IV drug abusers, and in patients with indwelling foreign bodies like pacemakers, catheters, or prosthetic joints [20]. Current treatment regime as per the infectious disease society of America (IDSA) is valve replacement with initial antifungal treatment with amphotericin B with or without flucytosine followed by long term suppression with fluconazole [21]. Patients who are poor surgical candidates should be placed in chronic lifelong suppression with fluconazole 400–800 mg (6–12 mg/kg) [18].

### 3.5. Surgical Management

Operative intervention should be considered in all individuals with the criterion mentioned in Table 3. Special consideration should be made to the clinical situation of the patient, and the likelihood of surviving the surgery on a case-to-case basis should be made. The main aim of surgery should be to eradicate infection and improve patient survival.

Mitral valve insufficiency precedes the development of heart failure and early intervention is recommended to improve survival. Antibiotic therapy alone does not lead to an improvement of mitral insufficiency [22]. The chances of a vegetation embolizing to distal circulation depend directly on the vegetation size. The chances of having an embolic phenomenon are as high as 70% in vegetation larger than 15 mm, in comparison to 27% in patients with size less than 15 mm. Similarly the mobile vegetation is much more likely to cause an embolism [23].

In case of a suspected embolic stroke, MRI scan of the brain is considered the most sensitive test for neurological imaging. Stroke can be seen in as much as 80% patients who have a left sided IE, on MRI scan. This MRI scan should be preceded by a noncontrast CT scan of the head to rule out bleeding; this may be secondary to a mycotic aneurysm and should warrant immediate neurosurgical evaluation. Patients with an active intracranial bleed are poor candidates for surgery [24, 25].

Splenic infarction is frequent in left sided IE and should not delay surgery. A CT scan of the abdomen and pelvis should be performed to rule out a splenic abscess. The missed diagnosis of a splenic abscess has grave consequences as it may cause infections of the new valve [26].  Table 4 summarizes the preoperative investigations for surgical approach.

The surgery should be timed as early as possible to avoid significant morbidity and improve survival. The main motive remains to avoid embolic phenomenon. There is limited data on the outcomes of valve surgery following infective endocarditis. A single center retrospective study noted that survival rates following surgery are acceptable with 30 day mortality at 8.5% and cumulative late mortality of 25.6% [27]. There is limited literature to comment on outcomes of valve surgery in cases with concurrent ESRD with IE.

## 4. Conclusion

Advances in medicine, public health, and economic developments have added an extra decade of life to the average human lifespan. Hemodialysis vascular access for initiation of hemodialysis has become crucial. Catheters are still common form of vascular access used for dialysis initiation due to late placement of AVF before dialysis, primary AVF failure, urgent initiation following acute kidney injury, unexpected decline in glomerular filtration rate, medical insurance issues, Surgeon shortage, and a lack of predialysis nephrology care. All dialysis units should take a multidisciplinary approach and have a referral process early on for access creation and avoid catheters and associated mortality. Patients who are not candidates for fistulas and grafts, as well as peritoneal dialysis, should be involved in a detailed goal of care discussion. The aim should be improving maximal care while compromising least on the quality of life.

## Figures and Tables

**Table 1:** High suspicion features mandating TTE in patients with HD and suspected IE.

High suspicion features for infective endocarditis mandating transesophageal echocardiogram after a TTE
(i) Patients with HD catheters
(ii) New onset congestive heart failure
(iii) Stigmata of endocarditis
(iv) HD related hypotension in a previously hypertensive patient
(v) Prior or repeated episodes of IE
(vi) Prior valvular surgery
(vii) Typical organisms for IE
(viii) Relapsing bacteremia after antibiotic discontinuation, regardless of causative pathogen

Gaetano et al., European heart journal (2007) 28, 2307–2312 doi:10.1093/eurheartj/ehm278.

**Table 2:** Depicting the suggested treatment regime for infective endocarditis in the general population as per guidelines published in American Family Physician.

Treatment regimen for Infectious endocarditis in general population	
Empiric therapy	(i) Vancomycin or ampicillin/sulbactam with an aminoglycoside (ii) Add rifampin in patients with prosthetic valves

Penicillin susceptible *viridans Streptococcus* or *Streptococcus bovis *(*S. bovis*)	(i) Penicillin G or ceftriaxone for 4 weeks *Or* (ii) Penicillin G plus gentamycin for 2 weeks *Or* (iii) Ceftriaxone plus gentamycin for 2 weeks *Or* (iv) Vancomycin for 4 weeks

Relatively penicillin resistant *viridans Streptococcus* or *S. bovis*	(i) Penicillin G or ceftriaxone for 4 weeks, plus gentamycin for 2 weeks Or (ii) Vancomycin for 4 weeks

Penicillin-resistant *viridans Streptococcus* or *S. bovis*	(i) Ampicillin plus gentamycin for 4–6 weeks Or (ii) Penicillin G plus gentamycin for 4–6 weeks Or (iii) Vancomycin for 6 weeks

Oxacillin- susceptible staphylococci	(i) Nafcillin or oxacillin for 6 weeks, plus gentamycin for 3–5 days (optional) Or (ii) Cefazolin for 6 weeks plus gentamycin for 3–5 days (optional)

Oxacillin-resistant staphylococci	(i) Vancomycin for 6 weeks

**Table 3:** Indication of surgical management of Mitral valve IE.

Indications for surgery in native valve endocarditis of mitral valve
(i) Moderate to severe or severe mitral regurgitation with or without heart failure
(ii) Vegetation size measuring more than 10 mm
(iii) Mobile vegetation
(iv) Paravalvular abscess
(v) Evidence of a single embolic phenomenon including stroke
(vi) Failure of antibiotic therapy
(vii) Infection with a fungal organism

[22].

**Table 4:** Suggested preop workup for surgical candidates.

Suggested preoperative work-up prior to considering surgery	

*Initial CT head*	(i) Embolic stroke (ii) Mycotic aneurysm (iii) Intracranial bleed

*MRI Brain*	(i) More sensitive for neuroradiological diagnosis

*CT chest, abdomen and pelvis*	(i) Pulmonary Infarcts (ii) Splenic and Hepatic Infarcts (iii) Splenic abscess contraindicating surgery

*Transesophageal echocardiography*	(i) More sensitive than TTE in visualization of vegetation (ii) Paravalvular Abscess

[22].
